# Higher growth variability and stronger responses to temperature changes in wild than hatchery‐reared sea trout (*Salmo trutta* L.)

**DOI:** 10.1002/ece3.7827

**Published:** 2021-07-14

**Authors:** Adam M. Lejk, Szymon Smoliński, Grzegorz Radtke, Andrzej Martyniak

**Affiliations:** ^1^ Department of Logistics and Monitoring National Marine Fisheries Research Institute Gdynia Poland; ^2^ Department of Fisheries Resources National Marine Fisheries Research Institute Gdynia Poland; ^3^ Department of Migratory Fish Inland Fisheries Institute in Olsztyn Żukowo Poland

**Keywords:** anadromous brown trout, Baltic Sea, Bayesian mixed‐effects models, fish, fish scales, sclerochronology

## Abstract

Each year, millions of hatchery‐reared sea‐run brown trout *Salmo trutta* L. (the sea trout) juveniles are released into the natural environment in the Atlantic region. The aim of this work was to investigate the growth responses of sea trout to changing temperature conditions and to compare the growth plasticity between wild and hatchery‐reared fish. Scales were collected from sea trout in a selected river flowing into the southern Baltic Sea. We analyzed the scale increment widths as a proxy of somatic growth and investigated the interannual variabilities and differences in growth between fish groups (wild and hatchery‐reared). We used mixed‐effects Bayesian modeling and ascribed the variances in growth to different sources. Furthermore, we developed indices of interannual (2003–2015) growth variation in the marine and freshwater phases of the life cycle of the fish and analyzed the relationships between trout growth and temperature. Temperature positively affects fish growth, regardless of the origin of the fish. We observed stronger relationships between fish growth and temperature conditions in the marine phase than in the freshwater phase. Additionally, wild sea trout are characterized by stronger responses to temperature variability and higher phenotypic plasticity of growth than those of the hatchery‐reared individuals. Therefore, wild sea trout might be better suited to changing environmental conditions than hatchery‐reared sea trout. This knowledge identifies possible threats in management actions for sea trout with an emphasis on ongoing climate change.

## Graphical abstract

Water temperature positively affects fish growth, regardless of the origin of the fish. Wild sea trout are characterized by stronger responses to temperature variability and higher phenotypic plasticity of growth than those of the hatchery‐reared individuals.

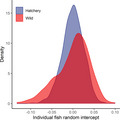

## 1 INTRODUCTION

The sea trout is an anadromous form of brown trout, *Salmo trutta* L., that naturally inhabits most of the northeast Atlantic basin (Elliott, 1994; Jonsson & Jonsson, 2011). The life history of this ecological form of the species is divided into freshwater and marine life stages, similar to the Atlantic salmon *Salmo salar* L. Juvenile sea trout stay in a riverine environment until the time of smolting and then start migrating to the sea. After this seaward migration, fish stay at sea for feeding and as adults return to their home river for spawning. Some individuals survive and return to the sea as *kelts*, feed intensively, and migrate again to spawn the next season (Elliott, 1994; Jonsson & Jonsson, 2011; Klemetsen et al., 2003). As a top predator, the sea trout has high ecological importance in aquatic ecosystems and is considered an indicator of good environmental status (HELCOM, 2011). Moreover, sea trout have high economic value for both recreational and commercial fisheries in freshwater and marine habitats (Blicharska & Rönnbäck, 2018; Milner et al., 2006; Solomon & Czerwinski, 2006).

Over recent decades, anthropogenic impacts and disturbances of the environment have negatively influenced sea trout populations in Europe (Jonsson & Jonsson, 2011; Nevoux et al., 2019). Overfishing, habitat degradation (e.g., dams and hydropower development, riverbed regulations), and water pollution are the main disturbances affecting sea trout populations (HELCOM, 2011). It is expected that ongoing climate change in the Atlantic region will strongly affect anadromous brown trout populations (Jonsson & Jonsson, 2009). Moreover, in the Baltic Sea region, overexploitation and an ulcerative dermal necrosis (UDN) disease generate high mortality rates in adult sea trout during the spawning run and negatively influence the status of sea trout populations (HELCOM, 2011; ICES, 2020).

While the freshwater stage of anadromous salmonids’ life is well studied (e.g., for Atlantic salmon and sea trout, see Elliott, 1994; Jonsson & Jonsson, 2011), there is less knowledge on their marine life stage (Eldøy et al., 2015; Flaten et al., 2016; Hansen & Quinn, 1998; Jensen et al., 2018). In particular, there is a deficiency of information regarding the environmental drivers of sea trout growth in the marine phase (e.g., Degerman et al., 2012; Fjørtoft et al., 2014; Olsen et al., 2006). Marine processes may be principal drivers of the population dynamics of anadromous fish species (Chaput, 2012; Davies et al., 2020; Pardo & Hutchings, 2020). Taking into consideration the changing environmental conditions of the global ocean (IPCC, 2019) and the general decreasing trend in the abundance of sea trout stocks (ICES, 2020; Nevoux et al., 2019), a more complete understanding of the marine life phase of this species is required.

Habitat degradation in spawning and nursery streams has resulted in a lower number of descending wild smolts (Jonsson & Jonsson, 2011). Consequently, compensatory stocking with hatchery‐reared fish has been applied for many populations. Stocking hatchery‐reared salmonid fish is one of the most commonly used fisheries management tools for the stock enhancement and recovery of wild populations around the world (Aprahamian et al., 2003; Bartel, 2001; Hay & Hatton‐Ellis, 2006; Poole et al., 2002). The differences between wild and hatchery‐reared fish in their responses to environmental conditions can influence their population dynamics and resistance (Araki et al., 2008; Kallio‐Nyberg et al., 1999, 2006). However, discrepancies in the plasticity of both groups are still poorly understood, and the long‐term consequences of these factors are hard to predict.

Plasticity determines how much the somatic growth rate can vary in response to environmental changes (Stawitz & Essington, 2019). Most fish species continue to grow throughout their lives, and most fish respond to changes in environmental conditions (e.g., improved feeding opportunities or shifts in temperature conditions) with changes in their growth rate (Jonsson & Jonsson, 2011). Therefore, fish growth might be a good indicator of ongoing alterations in a population, allowing the tracking of organisms’ responses to both direct and indirect environmental impacts (Rountrey et al., 2014). Variability in the growth of a fish species has further consequences for the fitness of individuals and whole populations reflected in, for example, fecundity, recruitment, or biomass (Daufresne et al., 2009). Thus, investigations of the drivers of fish growth variability are of high concern in ecological and fisheries research (Smoliński, 2019). In particular, the necessity of studies on marine growth has been previously emphasized, because marine growth drives the survival rates and recruitment rates of anadromous fish species—two parameters that are highly important for general population dynamics (Peyronnet et al., 2007).

Biochronology techniques use measurements of periodic increments (e.g., annual increments) formed on calcified structures (scales or otoliths) as a proxy of somatic growth (Brophy, 2014). Therefore, fish biochronologies may serve as indicators of the growth histories of individuals and may constitute a basis for the investigation of interannual variabilities or differences among groups (Hiilivirta et al., 1998; Lund & Hansen, 1991). Biochronology techniques may also be used for the identification of environmental factors that influence the growth of fish (e.g., Smoliński, 2019). Scale biochronologies also provide unique opportunities to study the growth of anadromous fish during their marine phase of life, affording insights that are logistically challenging to obtain using traditional methods, that is, direct body length measurements at sea or tag–recapture techniques. For example, previous studies applied analyses of scale growth patterns to test the hypothesis that marine growth modulates the survival rates of Atlantic salmon during the marine life stage (Friedland et al., 2005).

In this study, we used a collection of sea trout scales obtained from a selected river in the catchment area of the southern Baltic Sea. It is hypothesized that fish grow faster in warmer years (characterized by stronger biological production) and that wild fish have stronger growth plasticity in relation to temperature conditions than their hatchery‐reared counterparts. To address these research questions, we used mixed‐effects modeling and ascribed the observed variances in growth to different sources. Furthermore, with developed indices of interannual growth variation, we applied correlation analysis and tested the relationships between trout growth and temperature. The application of Bayesian techniques and integrated nested Laplace approximations allowed for the proper consideration of uncertainties in the model. Using this statistical approach, we investigated the growth responses of sea trout to changing temperature conditions in the marine and freshwater life phases and compared the growth plasticity of wild and hatchery‐reared fish.

## 2 MATERIALS AND METHODS

### 2.1 Study area

**FIGURE 1:**
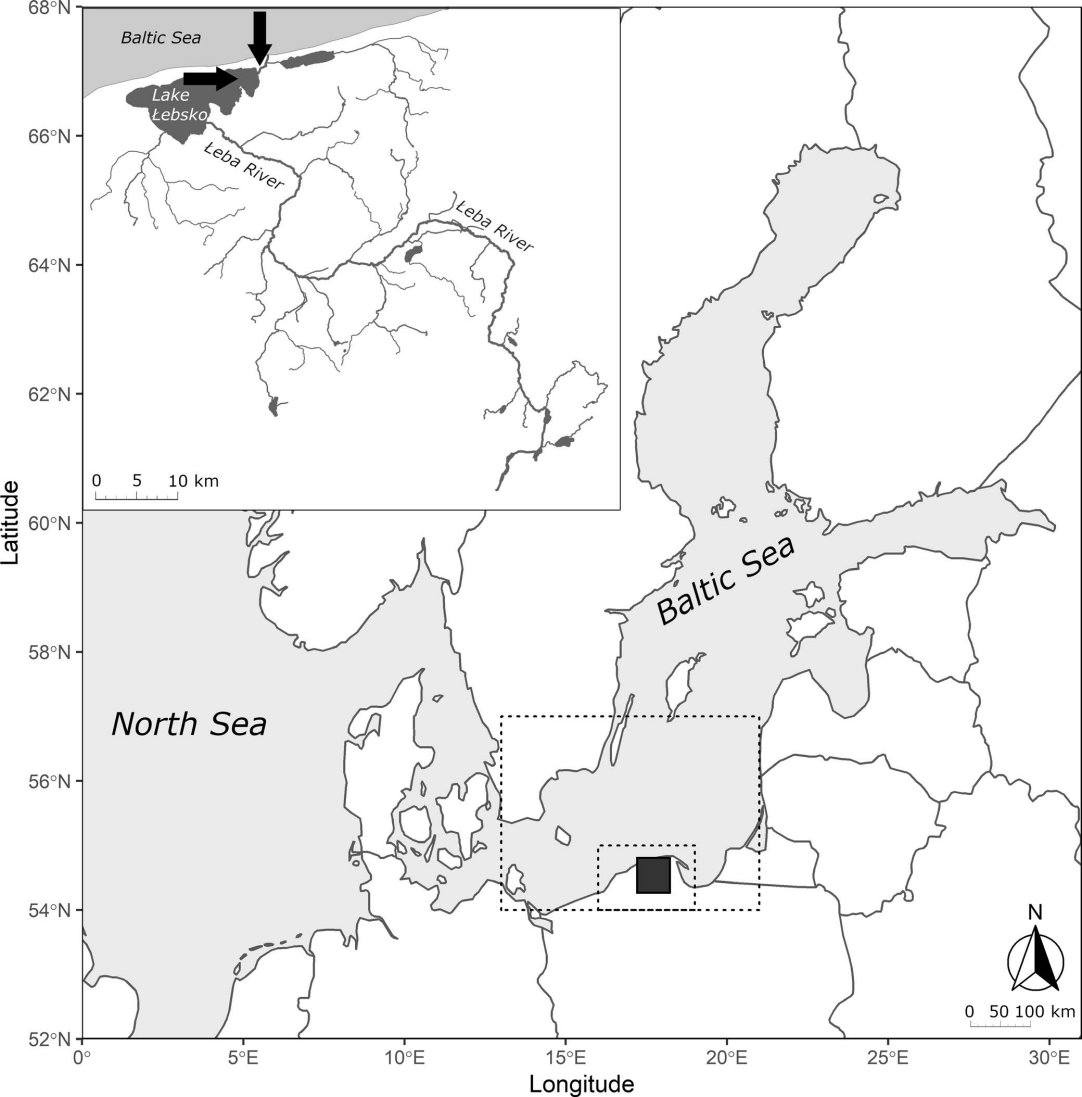
Map of the study area with the indicated regions over which the surface temperature data were aggregated (dashed rectangles). The area of the zoomed‐in map is marked with a black polygon. The horizontal arrow indicates the location of fish sampling. The vertical arrow indicates the smolts’ stocking site

The Baltic Sea is an epicontinental and semienclosed postglacial sea (Ojaveer et al., 2010; Figure 1). It is also one of the largest brackish water bodies in the world, with a mean depth of ca. 57 m and a maximum depth of 459 m (Snoeijs‐Leijonmalm & Andrén, 2017). The salinity regime is strongly determined by the amounts and frequencies of saline water inflows from the North Sea through the Danish Straits and freshwater inflows from rivers. The salinity gradient ranges from 2 g/kg in Bothnian Bay and in estuaries to 30 g/kg in the entrance to the North Sea (Ojaveer et al., 2010; Snoeijs‐Leijonmalm & Andrén, 2017). The Baltic Sea ecosystem is affected by a variety of human activities, for example, fisheries, maritime shipping, pollution, eutrophication, and habitat loss (Ojaveer et al., 2010; Reusch et al., 2018). The specific conditions of the sea have resulted in locally adapted populations of marine organisms (Reusch et al., 2018).

We studied a sea trout population from the Łeba River located in the southern basin of the Baltic Sea (northern Poland; Figure 1). The Łeba River is 126.7 km in length with a catchment area of 1,767.66 km^2^. The mean flow of the Łeba River in its lower course is approximately 11.7 m^3^/s. At the end of its course, the river passes through Lake Łebsko (71.42 km^2^), located in Słowiński National Park, which was included in the Natura 2000 European network of protected areas. Leaving the lake, the Łeba River carries its waters into the southern Baltic Sea (54º45’N; 17º33’E). This estuary section of the river is an area in which fish stocking with farmed smolts occurs (Figure 1). The main barrier that limits the occurrence of anadromous fish in the Łeba River is the weir of a small hydropower plant located 68 km from the river mouth.

### 2.2 Fish sampling

Sea trout were caught in Lake Łebsko (Figure 1) with the use of fyke nets during their spawning migration in autumn of each year from 2007 to 2016. The Łeba River basin is intensively stocked with sea trout alevins, fry, and 1‐year‐old smolts (ICES, 2020). Moreover, natural spawning takes place in the river system, mainly in small tributaries (Lejk & Martyniak, 2011). The stocking material is sourced from the native sea trout population originating from the Łeba River. In the period 2007–2016, all released smolts were adipose fin‐clipped; therefore, fin‐clipped individuals were classified composing the hatchery‐reared group, while individuals with the presence of an intact adipose fin were treated as the wild group (i.e., fish that smoltified in the wild). Therefore, hatchery‐reared fish stocked as alevins and fry were assigned to the wild group and their median contribution to the group was estimated at the level of ~24.1% (A.M. Lejk, *unpublished data*). Sea trout released in the younger stage as fry or alevins are influenced by the same processes as real wild fish (e.g., density‐dependent growth factors). Our approach is conservative because any potential genetic‐based differences in the studied thermal plasticity between wild and hatchery‐reared fish might be reduced by the hatchery‐reared fish stocked as alevins or fry, which were assigned to the wild group. We assumed that the spawners were caught in their natal river, demonstrating the homing phenomenon (Ferguson et al., 2019).

The caught adult fish were anesthetized in an 80 mg/L solution of MS‐222 (ACROS Organics^TM^, Belgium). The fork length (FL) of each fish was measured to the nearest 0.1 cm. Fish sex was recognized based on morphological features of each specimen. The presence or absence of the adipose fin was recorded. Samples of ca. 15–20 scales were collected from each fish for the determination of the age of the fish and for the further growth analysis. The scales were sampled 3–6 rows above the lateral line, between the dorsal and adipose fins (Elliott & Chambers, 1996). The scales were stored dry in paper envelopes and transported to the laboratory. In total, material was collected from 1,603 adult sea trout.

### 2.3 Fish scale analysis

Each scale was cleaned before the analysis by placing it in water for approximately one hour and drying thereafter. From the five to ten randomly sampled scales, only one scale with an easily visible and non‐regenerated nucleus was selected per individual for further analysis and placed between two microscope glass slides. The scales were analyzed using a Nikon AZ 100 M stereoscope microscope (Nikon Corp., Japan) at 20× and 80× magnifications to identify the position of the annual growth rings and each circulus. The freshwater and marine ages were determined based on the number of annuli (Shearer, 1992; Sych, 1967).

The age of each fish was described following the nomenclature and criteria reported by Elliott and Chambers (1996), wherein the first digit indicates the freshwater age (FW) prior to smolting and the second digit indicates the complete postmigration winters spent at sea (SW), which may be followed by a + indicating plus growth at the outer edge of the scale. In addition, spawning marks were identified from scale surface erosion (Elliott & Chambers, 1996).

The scales were measured using NIS‐Elements BR 3.1 computer software (Nikon Corp., Japan) at 20× magnification with reference to a calibrated scale bar, and the images were saved as high‐resolution TIFF images. We measured each annual growth zone (accuracy ± 0.01 μm) along a transect on the 360° axis of the scale, starting from the focus to the anterior edge of the scale and marking each annulus (Friedland & Reddin, 2000; Appendix 1). In some cases, it may be difficult to differentiate between run‐out and early sea growth due to dynamic changes in the environment of the sea trout during their seaward migration and partial feeding within rivers, estuaries, and coastal waters at the time of migration (Elliott, 1994; Jonsson & Jonsson, 2011; Klemetsen et al., 2003). Due to the possibility of scale regeneration during early growth (Marco‐Rius et al., 2013), there is a risk of incorrectly distinguishing freshwater and marine circuli (growth rings), especially for hatchery‐reared fish. To prevent these errors, we measured the first sea‐winter growth with the additional inclusion of a potential riverine period after the last freshwater winter. The circuli counts for each annual growth zone were made manually at 80x magnification, following the method described by Shearer (1992). Only circuli deposited on scales within an angle of 5° on each side of the defined measurement axis were counted.

### 2.4 Temperature data

Sea trout populations from various rivers of the Baltic Sea catchment show a variety of migration strategies. Tagging experiments have shown that the vast majority of individuals migrate short distances and that these migrations mainly occur in coastal waters, while other individuals undertake longer migrations (Bartel et al., 2001; Degerman et al., 2012; Kallio‐Nyberg et al., 2002), and a considerable percentage of sea trout from Polish rivers are caught far from the coast (Bartel et al., 2010). This phenomenon is observed especially for large sea trout individuals (Klemetsen et al., 2003). Above all, tagging studies have shown that the southern Baltic Sea is an important feeding ground for salmonid populations from around the Baltic Sea (ICES, 2020). Sea trout smolts enter the Baltic Sea mainly in April and May (Rasmussen, 1986; Rutkovska et al., 2019; del Villar‐Guerra et al., 2014, 2019). Therefore, to characterize the thermal conditions experienced by the fish at sea, we included in the model the mean surface temperature during the second half of the year (July–December) aggregated over the area from 54–57 °N–13–21 °E; the temperature data were obtained from the NOAA‐CIRES‐DOE Twentieth Century Reanalysis version 3 dataset, which provide temperatures modeled in a consistent way both for sea and land surface (Compo et al., 2011; Figure 1). Although the main aim of the analysis was to test the hypothesized effects of seawater temperatures on fish growth at sea, we also tested the potential thermal influences on fish growth in freshwater. It was assumed that in the studied river system, the surface and water temperatures are closely related (Smoliński & Glazaczow, 2019; Webb & Nobilis, 1997). Thus, the average annual surface temperature aggregated over the study area (54–55 °N–16–19 °E) was included in the analysis as a proxy of the water temperature experienced by the fish in freshwater. Temperature data were available for the whole analyzed fish growth period (2003–2015).

### 2.5 Data analysis

**FIGURE 2:**
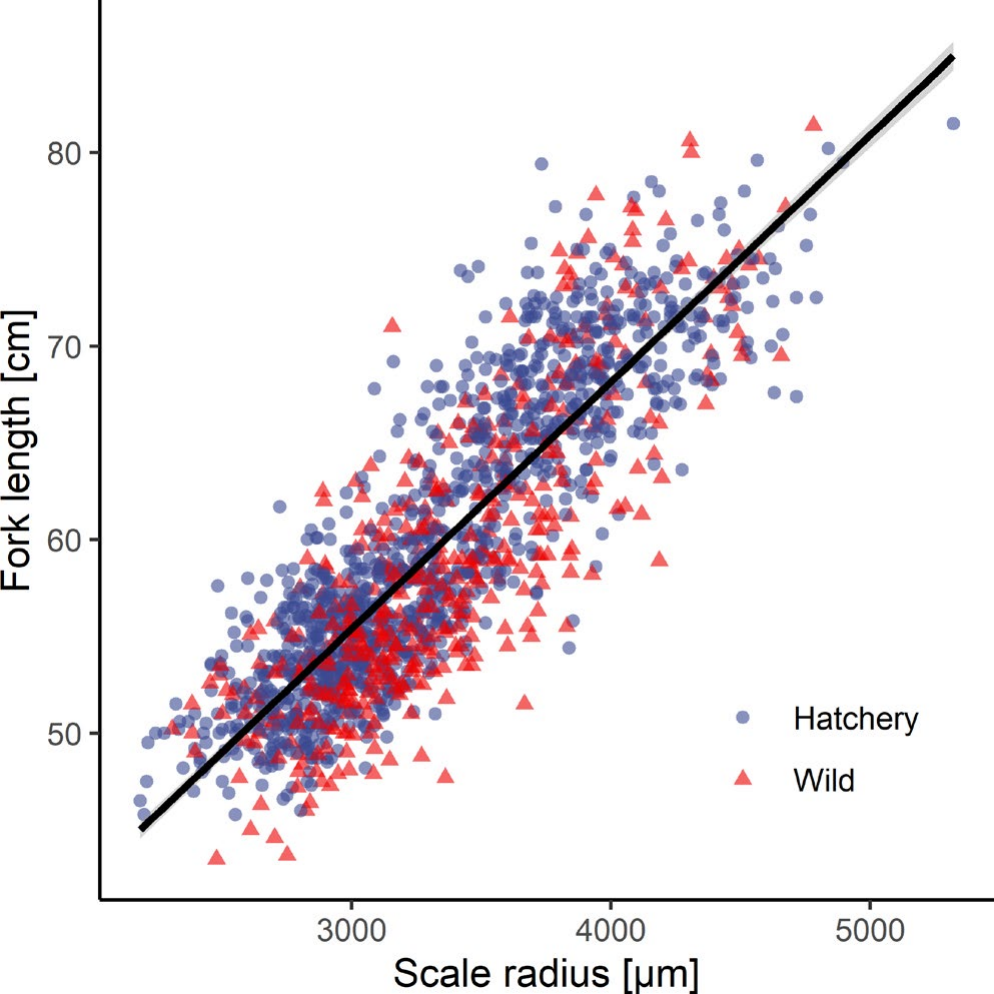
Relationships between the scale radius and fish fork length (FL). FL = 17.128 + 0.013 × scale radius, *F*_1,1594_ = 4,789, *R^2^* = 0.76, *p* < .001

From the 1,603 individuals caught in the years 2007–2016 (Appendix 2), we excluded fish with rare life histories (3 fish 2.0+, 3 fish 2.3+, and 1 fish 3.1+). We included measurements of scales from 1,596 fish (1,249 females, 347 males) in the analysis. We fitted two linear models with the fish fork length as a response variable and the scale radius or number of circuli as explanatory variables. In both models, we allowed for interactions between the given explanatory variables and the fish group: wild/hatchery (Figure 2, Appendix 3). This allowed us to check the assumptions of the relationships between fish somatic growth and the growth of scale (scale radius or the number of circuli) and to test for potential differences in these relationships between fish groups. Because the scale radius explained a higher proportion of the observed variance in fork length than the number of circuli, in further modeling, we used the width of the annual increment as a better proxy of fish somatic growth. Moreover, the width of each annual increment, as a continuous variable, assures a higher precision of the predicted growth than does the number of circuli, which is expressed as a discrete variable.

In the growth model, we excluded marginal scale increments (SW + age‐group, 28.7% of all increment measurements in the data set) since they do not reflect the growth of the whole year. We also omitted increments formed during spawning events in repeating spawners (2.96% of all increment measurements) because they do not represent the whole annual sea growth due to the scale erosion (Elliott & Chambers, 1996). In total, we included 3,808 increment measurements in the analysis.

We used linear mixed‐effects models to properly account for the repeated measurements of fish individuals and years and to partition the observed growth variabilities into interindividual and interannual sources (Weisberg et al., 2010; Morrongiello & Thresher, 2015; Smoliński et al., 2020). The width of each annual increment was treated as the response variable. We fitted the models with a random intercept for each fish, as well as group‐ and an environment‐specific random intercept for each year (Group:Environment:Year). We included the sex, age, and group of each fish as fixed effects, allowing an age–group interaction.

Bayesian techniques and integrated nested Laplace approximations were used to obtain model parameter estimates and predictions (Rue et al., 2009). To test the importance of the specified variables, models including different terms were compared with deviance information criterion (DIC) (Spiegelhalter et al., 2002). We assumed that the model term was supported if ΔDIC > 2. Penalized complexity priors were applied, but additional sensitivity tests were conducted with the default and half‐Cauchy priors (Simpson et al., 2017). Standard model diagnostics of the models were conducted to determine whether all assumptions were met (Zuur & Ieno, 2016). We used the R‐INLA package (Rue et al., 2009) and the R programming environment (R Development Core Team, 2018).

We extracted random intercepts for FishID from the growth model as a measure of growth plasticity at the individual level (not necessarily related to the temperature effects). Subsequently, we compared the variance in the random intercepts between groups (wild or hatchery) using the *F* test. To test for possible differences in thermal plasticity at the group level between wild and hatchery‐reared fish, we conducted additional correlation tests. The use of Bayesian techniques provides the posterior distribution of the estimates, in contrast to frequentist techniques that provide only point estimates (Hadfield et al., 2010; Houslay & Wilson, 2017). Therefore, we used posterior distribution of the year random effect estimates in further Monte Carlo correlation tests to properly account for the associated uncertainty. We randomly sampled the posterior results to visualize and statistically assess the relationships between mean annual growth (year random effects) and temperature.

## 3 RESULTS

We observed strong relationships between the fish fork length (mean ± standard deviation in wild fish = 59 ± 7.5 cm, hatchery‐reared = 60 ± 7.8 cm) and the radius of the scale (wild = 3,354 ± 484 μm, hatchery‐reared = 3,337 ± 537 μm; Figure 2). This relationship was consistent among both fish groups, as both the Group intercept and Radius*Group interactions were nonsignificant (Table 1). Similarly, we observed strong relationships between the fish fork length and the number of circuli (wild = 101 ± 12.4, hatchery‐reared = 101 ± 14.2; Appendix 3). Both the Group intercept and Circuli number*Group interactions were nonsignificant (Appendix 4). The linear model of fork length–radius explained 76% of the observed variance in fish somatic growth (Table 1). The model for the length–circuli number relationships explained 74% of the variance (Appendix 4).

**TABLE 1:** Summary statistics of the fork length–scale radius relationship models

Predictor	Estimate	CI	*p*‐value
Intercept	17.55	16.17–18.94	<.**001**
Radius	0.01	0.01–0.01	<**.001**
Group [Wild]	−1.78	−4.61–1.04	.216
Radius * Group [Wild]	0.00	−0.00–0.00	.767
Observations	1596		
*R^2^*	0.76		

Notes

CI, confidence intervals; *p*, significance level.

Significant *p*‐values (< .001) are in bold.

The estimations of age and interpretations of the scale patterns indicated that, of the 1596 selected fish, most of the trout migrated after one year from the freshwater to the sea (89.9%), while the rest (10.1%) spent two years in the freshwater before their seaward migration. Most of the fish (63.0%) spent one year at sea, while 35.1% spent two and 1.9% spent three years at sea, before being caught in Lake Łebsko. Among the selected sea trout individuals, 10.7% were repeat spawners.

**FIGURE 3:**
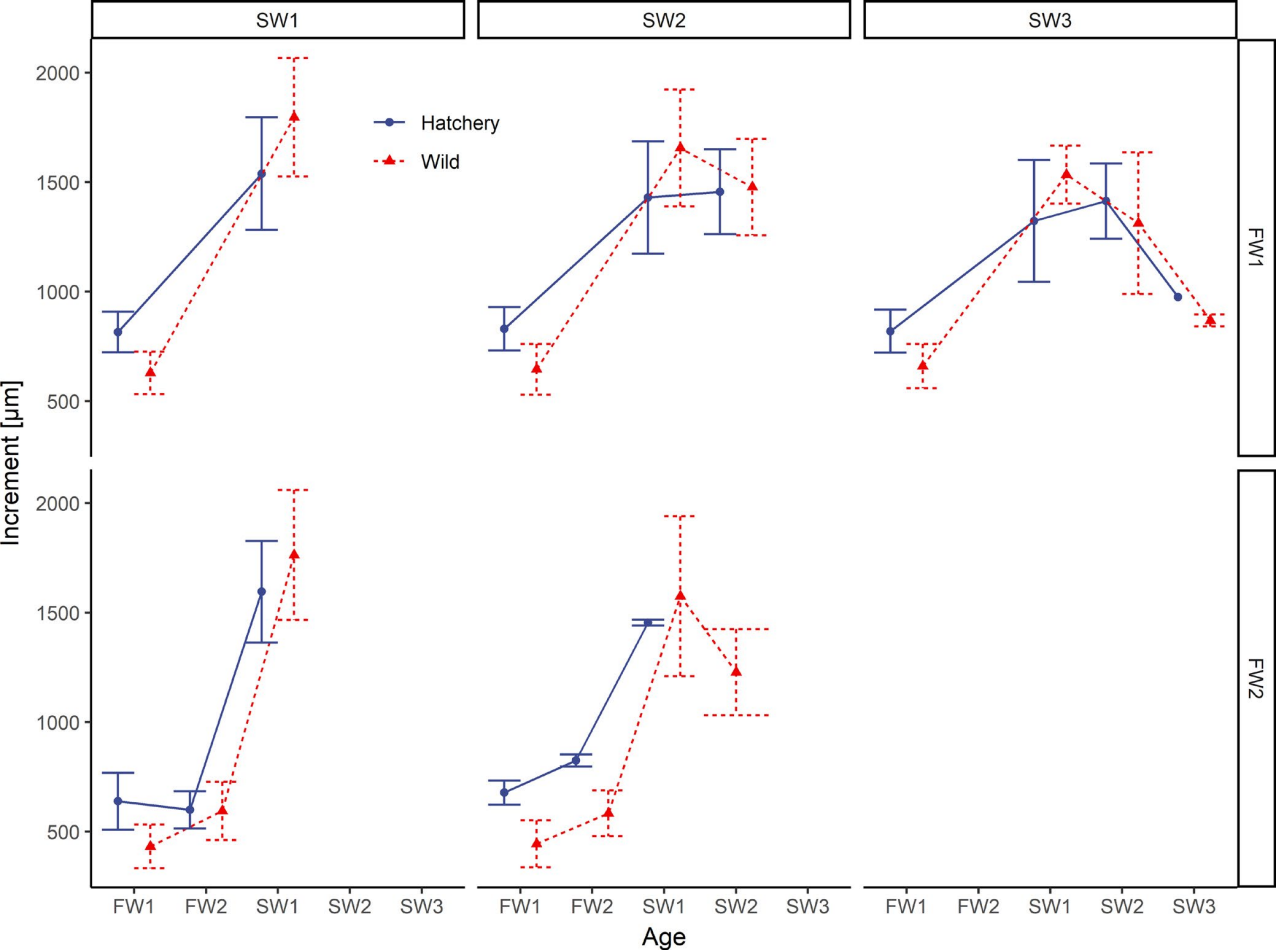
Measurements of the growth increments of fish scales. Points and error bars indicate mean ± standard deviation. Fish are grouped by sea age (columns) and freshwater age (rows). The vertical line shows the transition from freshwater to marine ecosystems, and “FW” and “SW” on the *x*‐axis indicate the freshwater and sea age, respectively. SW + increments and increments formed during spawning events in repeating spawners were excluded from the plot

The measurements of the annual increments of scales showed that the growth that occurred during the second year of the freshwater phase (if an individual did not migrate after one season toward the sea) was, on average, slightly slower in hatchery‐reared fish and more intensive in wild fish relative to the growth in the first year (Figure 3). Among these individuals, the growth of their scales during the first year was, on average, 645 ± 120 μm and 435 ± 101 μm for hatchery‐reared and wild groups, respectively. During the second year, the growth measurements were 638 ± 117 μm and 593 ± 127 μm for the hatchery‐reared and wild groups, respectively. In general, sea growth was more intensive than freshwater growth. Wild fish that entered the sea as FW1 smolts grew faster (1,751 ± 276 μm) at sea than hatchery‐reared FW1 smolts (1,492 ± 263 μm) in the first year at sea (Figure 3). There was a considerable variation of growth trajectories between fish individuals (Appendix 5). Sea growth decreased with fish age; on average, sea growth was 1,563 ± 293 μm in the first year at sea, 1,445 ± 205 μm in the second year, and 904 ± 65 μm in the third year.

**FIGURE 4:**
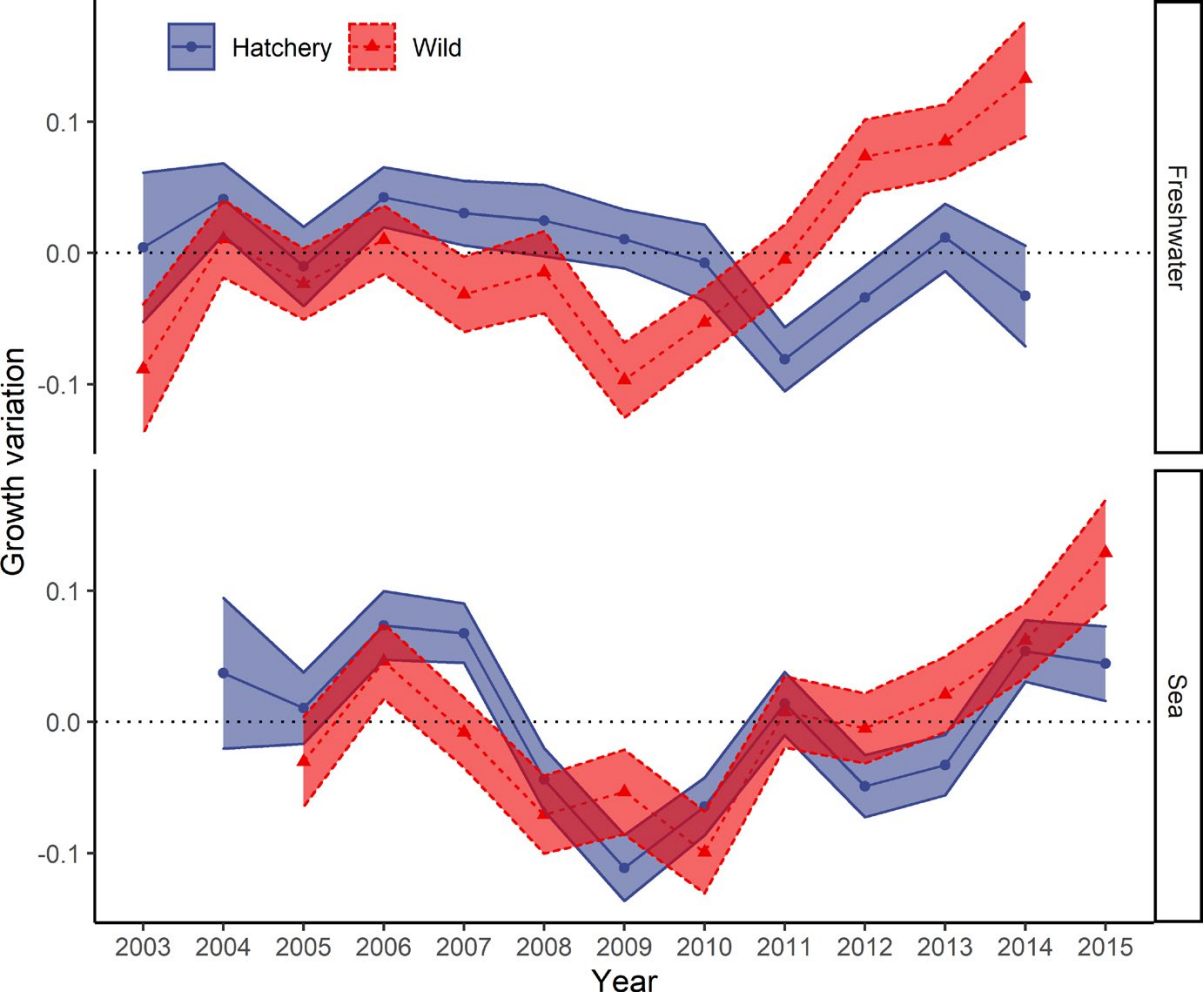
Interannual growth variation in sea trout in the freshwater (upper panel) and sea (lower panel) stages. The time series represent the posterior distribution (mean ± standard deviation) of the year random effects of the selected growth model

We observed interannual variation in sea trout growth in both the freshwater and marine phases (Figure 4). The random year intercepts for each group of fish and environment explained 13% of the variance (Table 2). The posterior distribution of the year random effects obtained from the growth model indicated that freshwater growth was more stable over time for hatchery‐reared fish and more fluctuating for wild fish. There was also a noticeable interannual variation in the sea growth of hatchery‐reared and wild fish. For the wild group, long‐term positive trends were observed for both freshwater and sea growth, with the highest values observed in the most recent years (2014 and 2015, respectively).

**TABLE 2:** Growth model parameter estimates

Predictor	Estimate	CI
Intercept	6.70	6.66–6.74
Age [FW2]	−0.23	−0.33–−0.14
Age [SW1]	0.61	0.55–0.67
Age [SW2]	0.58	0.52–0.63
Age [SW3]	0.23	−0.09–0.56
Group [Wild]	−0.39	−0.44–−0.33
Age [FW2] * Group [Wild]	0.32	0.22–0.42
Age [SW1] * Group [Wild]	0.53	0.44–0.61
Age [SW2] * Group [Wild]	0.35	0.26–0.44
Age [SW3] * Group [Wild]	0.24	−0.16–0.64
Random Effects		
*SD* for the Gaussian observations	0.155	
*SD* for FishID	0.058	
*SD* for Group:Environment:Year	0.065	

Note

CI, confidence intervals of the posterior distribution, *SD*, standard deviation associated with the random effect.

**FIGURE 5:**
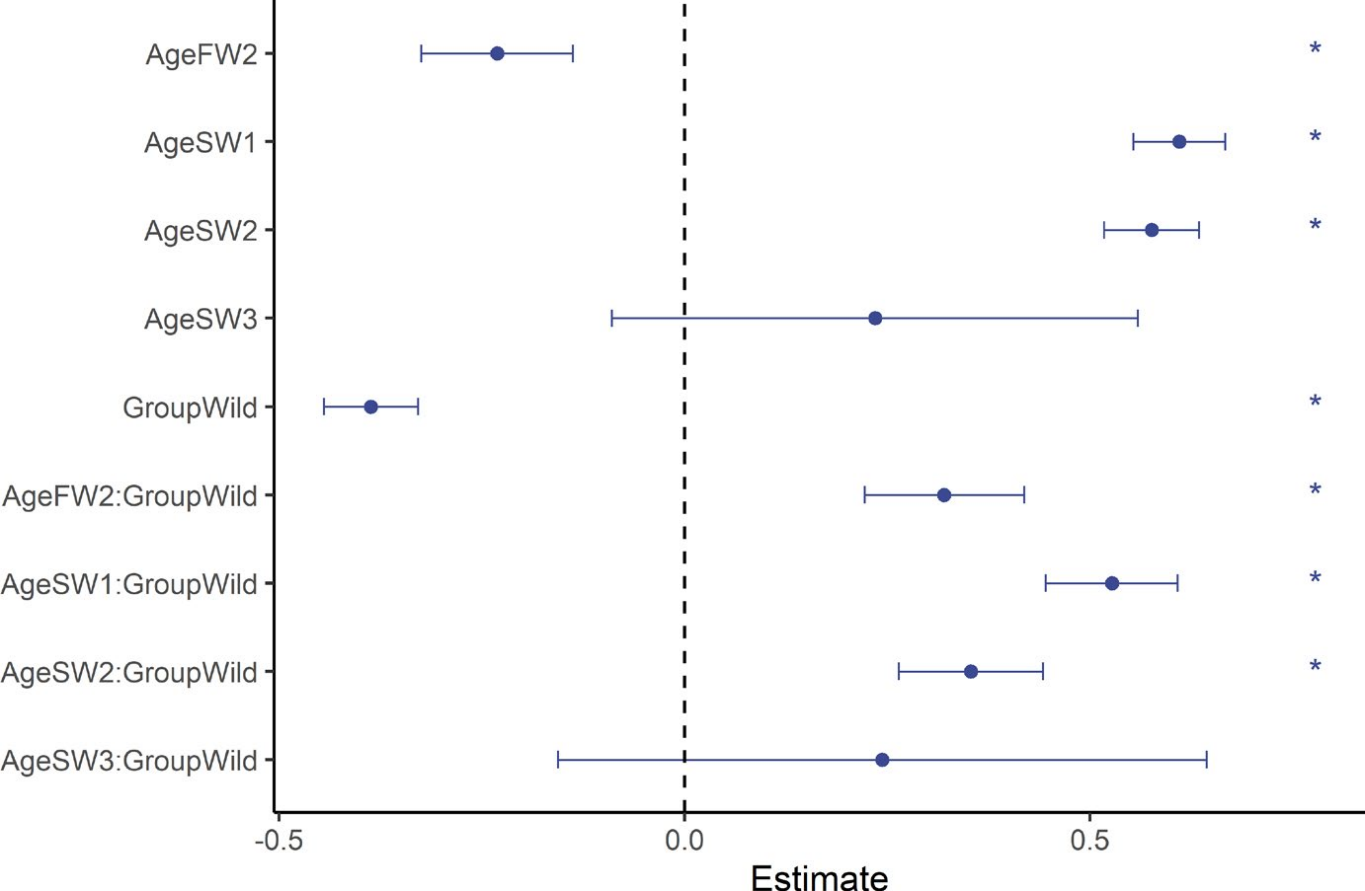
Posterior distribution of the growth model parameter estimates obtained with the Bayesian technique and integrated nested Laplace approximations. The points show the mean estimates, the bars indicate the confidence intervals, and the stars show effects that are considered important (when the 95% confidence interval (CI) of the parameter estimates does not overlap zero). For better visibility, estimates of the intercepts are omitted (6.70; CI = 6.66–6.74)

Based on the model comparisons, we found no support for the effect of sex (ΔDIC < 2; Appendix 6), and this term was dropped at the preliminary phase. There were, however, important effects caused by the fish age and group (wild or hatchery‐reared), as well as the interaction between these two variables (Table 2, Appendix 7, Figure 5). The model estimates indicated that sea growth was more intensive than freshwater growth and that wild fish grew more than their hatchery‐reared counterparts (Table 2, Figure 5). There was a high uncertainty in the parameter estimates regarding growth for the age‐group SW3 due to the low number of observations. Estimates of the fixed effects were insensitive to the priors applied (Appendix 8).

**FIGURE 6:**
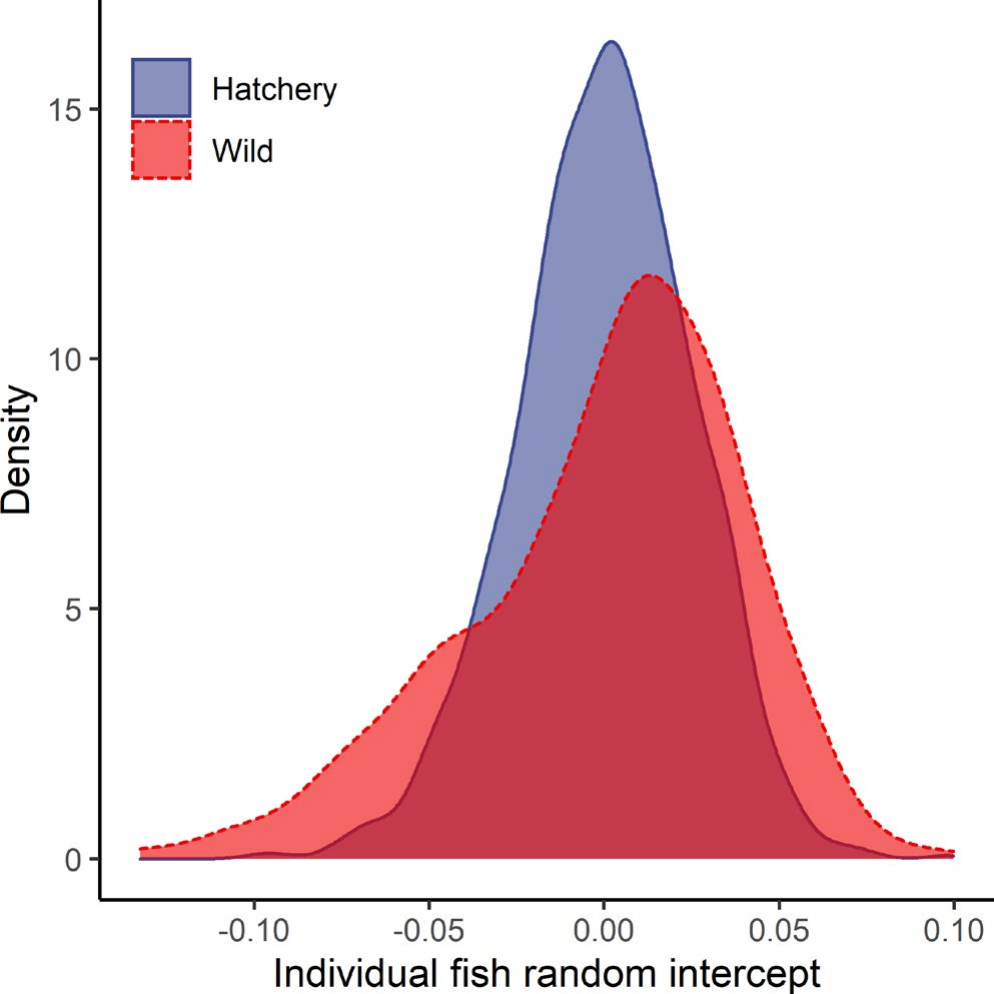
Distribution of the individual fish random intercepts estimated by the growth models

The random FishID intercepts explained 11% of the variance (Table 2). Estimates of the random effects were insensitive to the priors applied (Appendix 9). A difference was observed in the distribution of the individual fish random intercepts estimated in the growth model between wild and hatchery‐reared trout (Figure 6). The conducted test indicated a ratio of variances (variance in hatchery‐reared fish to variance in wild fish) at the *F* = 0.41 level (numerator *df* = 1,146, denominator *df* = 448, *p* < .001).

**FIGURE 7:**
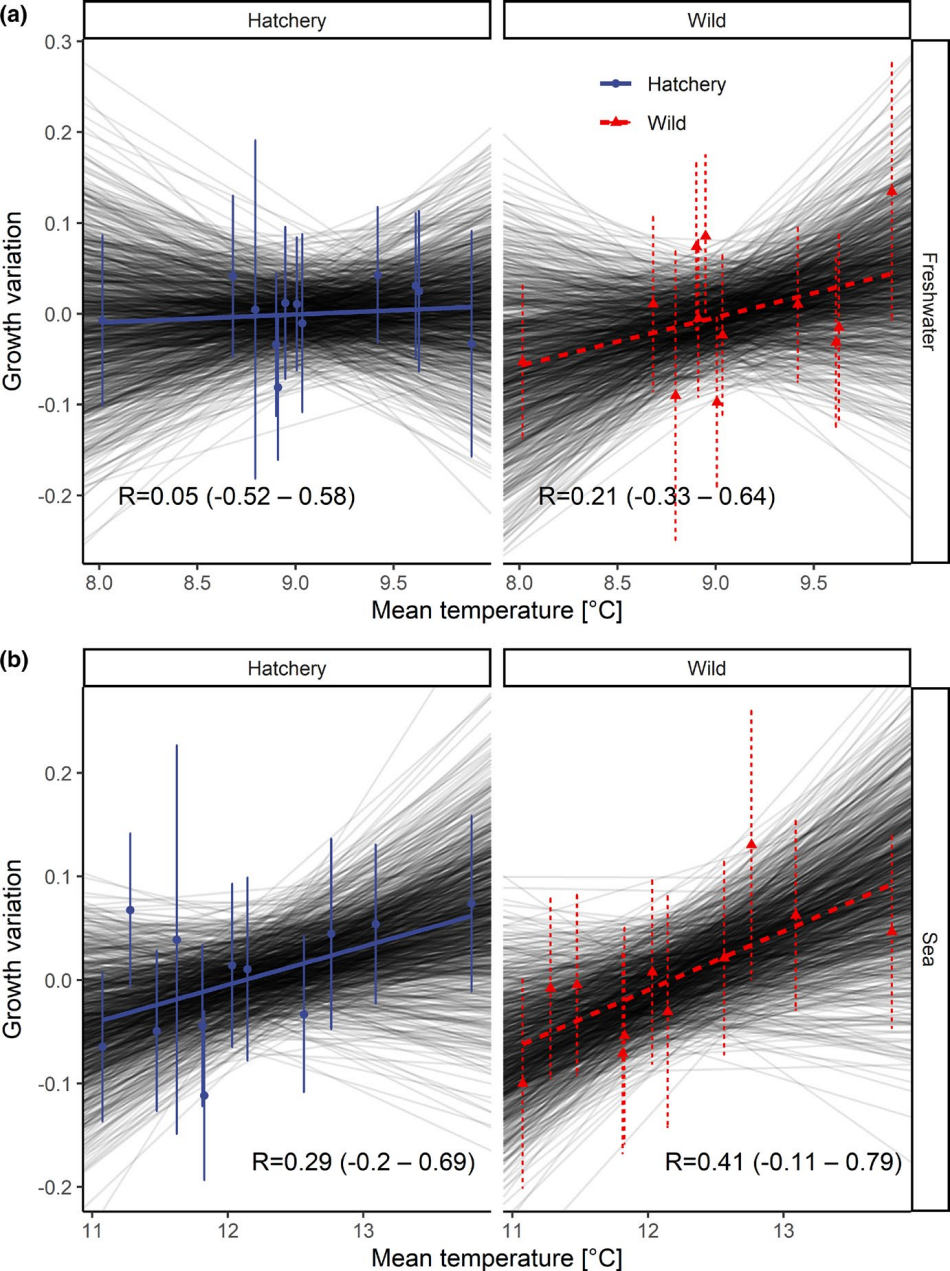
Correlations between freshwater (a) and sea (b) mean growth time series and mean monthly surface (skin) temperatures for both environments. Mean correlations are given with 95% confidence intervals (CIs). The points and error bars represent the means and standard deviations of the posterior distribution of the random year effects (index of growth variability). The transparent thin lines reflect 1,000 models obtained from Monte Carlo simulations considering uncertainty in the growth variation estimates using posterior distribution. The bolded thick lines represent the models fitted to the mean values of the posterior distribution

The mean surface temperature during the second half of the year (July–December) for the sea environment (aggregated over the area from 54–57 °N – 13–21 °E) varied from 11.1°C to 13.8°C (long‐term average = 12.0 ± 0.7°C). The mean annual surface temperature aggregated over the freshwater study area (54–55 °N – 16–19 °E) varied from 8.0°C to 9.9°C (long‐term average = 9.1 ± 0.5°C). No important relationships between freshwater mean growth and mean monthly surface water temperatures were found for hatchery‐reared fish (*r* = 0.05; Figure 7), whereas there was a higher correlation determined between freshwater growth and temperature for wild trout (*r* = 0.21). Stronger relationships than those obtained for freshwater growth were found between sea growth and temperature for both hatchery‐reared and wild fish (*r* = 0.29 and *r* = 0.41, respectively). However, Monte Carlo simulations that considered uncertainty in their growth variation estimates (using posterior distribution) showed high uncertainty in the results of the correlation test in all cases (95% confidence intervals overlap zero).

## 4 DISCUSSION

We observed lower growth in the second year of life in hatchery‐reared fish than in wild fish. This may be caused by equal conditions in the hatchery, the genetic background of the fish, persistent phenotypic effects (Weber & Fausch, 2003), or behavioral differences between wild and hatchery‐reared fish, for example, problems exploiting natural prey (Vehanen et al., 2009). The stocking of fish in river mouths or estuaries is often practiced to minimize early mortality (Jonsson et al., 1994; Cowx, 1994). Most fish released as 1‐year‐old smolts in the lower part of a river generally run out to the sea within a few days and are not influenced by changing conditions in the river environment (Hansen et al., 1989). The exceptions are individuals that stay in the freshwater environment for an additional winter. Low numbers of these overwintering hatchery‐reared fish may influence the representativeness of our data and the accuracy of our estimates, but we assume that observed lower growth could be the consequence of the lower adaptation of the hatchery‐reared individuals to the natural environment. On the other hand, since the growth rate in trout is typically inversely related to size, wild individuals that obtained smaller sizes at the end of the particular year may grow more in the consecutive years through the mechanisms of compensatory growth (Ricker, 1975). Thus, variation in fish size at hatching, and variation in fish size at first entry to sea observed between groups contribute to the differences in growth rates during their later life.

We observed that wild fish that spent two years in freshwater grew faster in the second year than in the first year in this zone. This could be explained by dietary shifts from drifting invertebrates toward small fish and amphibians that are more caloric and accelerate somatic growth (Elliott, 1994; Jonsson & Jonsson, 2011). In addition, during their second year of life in a freshwater system, competition is reduced among juvenile salmonids because part of their cohort has already migrated toward the sea (Klemetsen et al., 2003) or is widely dispersed within the nursery stream (Elliott, 1994; Lejk & Radtke, 2021).

In biochronological studies, year random effects are often used to represent the systematic deviances in growth from the overall average that are attributed to the combined extrinsic environmental effects (Smoliński et al., 2020). Typically, point estimates of these effects are obtained with the best linear unbiased predictors using a frequentist framework (but see, e.g., the application of Bayesian methods for Pacific geoduck growth in Helser et al., 2012). The obtained estimates are then often applied in the second phase of statistical tests (Houslay & Wilson, 2017). A widely neglected fact is that errors that are inherent in the prediction of random effects based on the best linear unbiased predictors can “propagate” to further statistical tests and are not correctly accounted for in the secondary statistical tests. Consequently, these secondary statistical tests are nonconservative (Hadfield et al., 2010; Houslay & Wilson, 2017). Here, our correlation tests are based on the posterior distribution obtained with integrated nested Laplace approximations. Such an approach allows proper accounting for uncertainty in the random effect estimates.

Our results demonstrate that hatchery‐reared sea trout show more equal interannual growth associated with the same initial rearing conditions in the hatchery. In contrast, wild trout juveniles are influenced by a range of environmental factors. Both spatial and temporal differentiations in ambient environmental conditions are typically observed for this fish species during the freshwater phase (Elliott, 1994; Jonsson & Jonsson, 2011; Klemetsen et al., 2003). Different origins of fish may influence their growth totals in the natural environment and their level of phenotypic plasticity (Weber & Fausch, 2003). This phenomenon was visible in the more spread‐out estimates of the random year effects for the freshwater growth of wild fish when compared to those of their hatchery‐reared counterparts.

The phenotypic plasticity of growth at the individual level investigated here is reflected by variation in the average scale increment width in the studied fish. Our results highlighted the lowered variance in random intercepts for fish individuals of the hatchery‐reared group in contrast to those of the wild group. Equal conditions at the beginning of the lifetimes of the hatchery‐reared fish might reduce fish plasticity and the range of average individual body size. In addition, stable hatchery conditions may result in the failure of fish to develop a physiological response capacity (Araki & Schmid, 2010). This could also be the result of a more similar genetic background among hatchery‐reared fish determining the plasticity of individuals (Hutchings, 2004; Rogell et al., 2012). If the variability in traits is lowered, it may reduce the capacity for evolutionary selection and the ability of the species to adapt to changing environments, a phenomenon that is crucial for maintaining species continuity under changing conditions (Hutchings, 2004).

Among the biological parameters, the sea growth of anadromous salmonids is strongly influenced by water temperatures in marine environments and has been widely investigated for Atlantic salmon (Friedland et al., 2000, 2005) but not for sea trout. Our findings show that sea growth has a stronger relationship with surface temperature than does freshwater growth. Temperature effects on fish growth were observed for both groups, but wild fish responded more intensively both in freshwater and in the sea, than hatchery‐reared individuals. These findings support previous conclusions that wild individuals might be better suited to the environment and might be characterized by higher growth plasticity in response to changing seawater temperature (Araki & Schmid, 2010; Vehanen et al., 2009).

The temperature data used in this study represent only a proxy of the fish growth conditions that exist in freshwater and marine environments. Despite the potential discrepancies between the applied variables and temperatures experienced by fish, we were able to detect relationships between temperature conditions and fish growth. Temperature is one of the main factors affecting biological processes in marine ecosystems, and thermal conditions also affect sea trout growth (Kristensen et al., 2018). Temperature strongly regulates fish metabolism, which is linked with somatic growth and oxygen consumption (Currie & Schulte, 2014). Climate, in addition to its direct effects, may affect fish growth indirectly by influencing other organisms, modulating food availability, and intra‐ or interspecific interactions (Friedland et al., 2005; Jonsson & Jonsson, 2011; Rolls et al., 2017).

Since the effect of temperature on fish growth was one of the main interests in this study, no other environmental variables were taken into account. In the freshwater phase, other physical parameters, such as river flow variation (Elliott, 1994; Jonsson & Jonsson, 2011) and spatial heterogeneity, for example, shelter availability (Finstad et al., 2009), can be even more important for fish growth than temperature. Additionally, density‐dependent mechanisms affect salmonid fish growth and mortality during their early life stages (Lobón‐Cerviá, 2007). Fish density is directly linked with mutual competition and food availability (Elliott, 1994). Negative density dependence in sea trout growth is a less important factor in marine environments than in freshwater environments (Marco‐Rius et al., 2013). Nevertheless, it is advised that other factors, for example, oceanographic or large‐scale climatic conditions (e.g., Friedland et al., 2005), be integrated in future investigations of sea trout plasticity.

Strong anthropogenic pressures on the environment and aquatic living resources force activities that compensate for the damages caused. Fish stocking is an active form of restoration and enhancement for many fish populations and is a compensatory function for overfished resources (Aprahamian et al., 2003; Cowx, 1994). However, the stocking and introduction of hatchery‐reared fish, which are characterized by some level of domestication and reduced genetic pool due to the limited number of parental individuals, are very controversial (Bernaś et al., 2014; Teletchea & Fontaine, 2014; Wang et al., 2002). The abilities of stocked fish to adapt to local environmental conditions and their ecological impacts have been widely shown (e.g., Araki & Schmid, 2010; Bolstad et al., 2017; Jonsson & Jonsson, 2006). As a result, these factors may cause reductions in genetic diversity in stocked populations (Araki et al., 2008). Furthermore, the rearing process has a negative effect on the fitness of stocked fish (Araki & Schmid, 2010). There is also a range of differences between wild and hatchery‐reared fish, the consequences of which are difficult to predict. For example, hatchery‐reared and wild Atlantic salmon can show distinct migratory behaviors (Jutila et al., 2003; Salminen et al., 1994), such as the timing of their ascents to rivers (Fleming et al., 1997; Jonsson et al., 1994), different spawning peaks (Lura & Sægrov, 1993), and different rates of straying to foreign rivers (Degerman et al., 2012; Jonsson et al., 2003). Moreover, adult sea trout originating from farmed smolts released in a river mouth reached the spawning grounds located in the upper river course less frequently than wild individuals (Dębowski et al., 2011). Additionally, our results indicate the reduced growth plasticity of hatchery‐reared fish in response to changing temperatures. These discrepancies in genetic diversity, behavior, and growth can have further implications for population dynamics and resistance to environmental changes, especially climatic changes.

Our results show that temperature affects fish growth, regardless of the origin of the fish. However, wild sea trout stand out by their stronger responses to temperature variability and their higher phenotypic plasticity of growth than those of hatchery‐reared individuals. Therefore, wild sea trout, through their elevated phenotypic plasticity, might be better suited and more resistant to changing environmental conditions than hatchery‐reared sea trout. Stocking with hatchery‐reared fish may deplete this adaptive capacity and result in stock degeneration. This knowledge identifies possible threats in management actions for sea trout. Our results support the promotion of natural reproduction in conservation programs.

## CONFLICT OF INTEREST

The authors have no conflicts of interest to declare.

## AUTHOR CONTRIBUTION

**Adam M. Lejk:** Conceptualization (lead); Investigation (lead); Project administration (lead); Writing‐original draft (lead); Writing‐review & editing (lead). **Szymon Smoliński:** Conceptualization (lead); Formal analysis (lead); Methodology (lead); Writing‐original draft (lead); Writing‐review & editing (lead). **Grzegorz Radtke:** Investigation (supporting); Writing‐review & editing (supporting). **Andrzej Martyniak:** Supervision (supporting); Writing‐review & editing (supporting).

## Data Availability

Data have been deposited in Dryad Digital Repository at https://doi.org/10.5061/dryad.3n5tb2rht.

## APPENDIX 1

Example of a sea trout scale aged 2.1+ (FL = 58.9 cm, body weight = 2,235 g) collected in November 2014, showing the measurement axis with particular growth increments

## APPENDIX 2

The numbers of wild and hatchery‐reared sea trout sampled in Lake Łebsko in the period 2007–2016

## APPENDIX 3

Relationships between the circuli number and fish fork length (FL). FL = 11.231 + 0.481 × circuli number, *F*_1,1594_ = 4,413, *R^2^* = 0.74, *p* < .001

## APPENDIX 4

Summary statistics of the fork length–circuli number relationship models. CI, confidence intervals; *p*, significance level

**Table:**

Predictor	Estimate	CI	*p*‐value
Intercept	12.01	10.37–13.65	**<.001**
Circuli number	0.48	0.46–0.49	**<.001**
Group [Wild]	−2.98	−6.40–0.44	.088
Circuli number * Group [Wild]	0.02	−0.01–0.05	.234
Observations	1596		
*R^2^*	0.74		

## APPENDIX 5

Measurements of the growth increments of fish scales. The line paths represent the growth histories of fish individuals. Fish are grouped by sea age (columns) and freshwater age (rows). The vertical line shows the transition from freshwater to marine ecosystems, and “FW” and “SW'' on the x‐axis indicate the freshwater and sea age, respectively. SW+ increments and increments formed during spawning events in repeating spawners were excluded from the plot

## APPENDIX 6

Results of the model comparison. ΔDIC is the difference in the deviance information criterion between the particular model and the best model, and “*” indicates the interaction between terms

**Table:**

Fixed effects	DIC	ΔDIC
Age* Group	−2942.125	0
Age* Group + Sex	−2940.671	1.453
Age* Group + Sex* Group	−2938.606	3.518

## APPENDIX 7

Results of the model comparison. ΔDIC is the difference in the deviance information criterion between the particular model and the best model, and “*” indicates the interaction between terms

**Table:**

Fixed effects	DIC	ΔDIC
Age	−2837.368	104.7567
Group	−2723.764	218.3611
Age + Group	−2838.588	103.5363
Age* Group	−2942.125	0

## APPENDIX 8

Results of the sensitivity tests conducted with different priors. The points indicate the mean, and the error bars indicate the mean ± *SD* of the posterior distribution for the fixed effects included in the growth model

## APPENDIX 9

Results of the sensitivity tests conducted with different priors. The points indicate the mean, and the error bars indicate the mean ± *SD* of the posterior distribution for the random effects included in the growth model
